# Diffusion-weighted imaging and dynamic contrast-enhanced MRI in assessing response and recurrent disease in gynaecological malignancies

**DOI:** 10.1186/s40644-015-0037-1

**Published:** 2015-03-15

**Authors:** Ayshea Hameeduddin, Anju Sahdev

**Affiliations:** Imaging Department, St. Bartholomew’s Hospital, Barts Health, West Smithfield, London, UK

**Keywords:** Adnexal, Functional MRI, Diffusion weighted imaging, Dynamic contrast enhancement, Cervical carcinoma, Endometrial carcinoma, Ovarian carcinoma

## Abstract

Magnetic resonance imaging (MRI) has an established role in imaging pelvic gynaecological malignancies. It is routinely used in staging endometrial and cervical cancer, characterizing adnexal masses, selecting optimal treatment, monitoring treatment and detecting recurrent disease. MRI has also been shown to have an excellent performance and an evolving role in surveillance of patients after chemoradiotherapy in cervical cancer, post-trachelectomy, detecting early recurrence and planning exenterative surgery in isolated central recurrences in both cervical and endometrial cancer and in young patients on surveillance for medically managed endometrial cancer. However, conventional MRI still has limitations when the morphological appearance of early recurrent or residual disease overlaps with normal pelvic anatomy or treatment effects in the pelvis. In particular, after chemoradiotherapy for cervical cancer, distinguishing between radiotherapy changes and residual or early recurrent disease within the cervix or the vaginal vault can be challenging on conventional MRI alone. Therefore, there is an emerging need for functional imaging to overcome these limitations. The purpose of this paper is to discuss the emerging functional MRI techniques and their applications in predicting treatment response, detecting residual disease and early recurrent disease to optimize the treatment options available using diffusion-weighted imaging and dynamic contrast enhancement particularly in cervical and endometrial cancer.

## Introduction

MRI has a pivotal and established role in detection and staging of gynaecological malignancy. The exquisite soft tissue resolution of MRI allows accurate demonstration of tumour size, location, extension and nodal involvement. Despite excellent clinical utilisation to date, conventional T1 and T2 sequences cannot provide information about tumour microenvironment and have limitations in assessing response of tumours to therapy and in particular, differentiating residual or recurrent disease from post-treatment fibrosis due to overlap of morphological appearances [1]. This latter distinction is crucial in selecting patients who may benefit from further salvage treatment options.

Functional MRI has evolved over recent years with the development of stronger field strengths, receiver coils and pulse sequences and has proven benefit in cerebral, breast and rectal cancers [2]. The clinical utilisation of functional MRI in gynaecological malignancy is not yet established. There is however an emerging body of evidence to support its use in the assessment of tumour response to therapy with promising results to date, particularly in cervical cancer.

As we learn more about tumour heterogeneity and develop sophisticated therapeutic options, the need for an imaging biomarker is essential to improve patient care and ultimately long-term survival.

## Review

### Clinical need for functional imaging techniques

**Figure 1:**
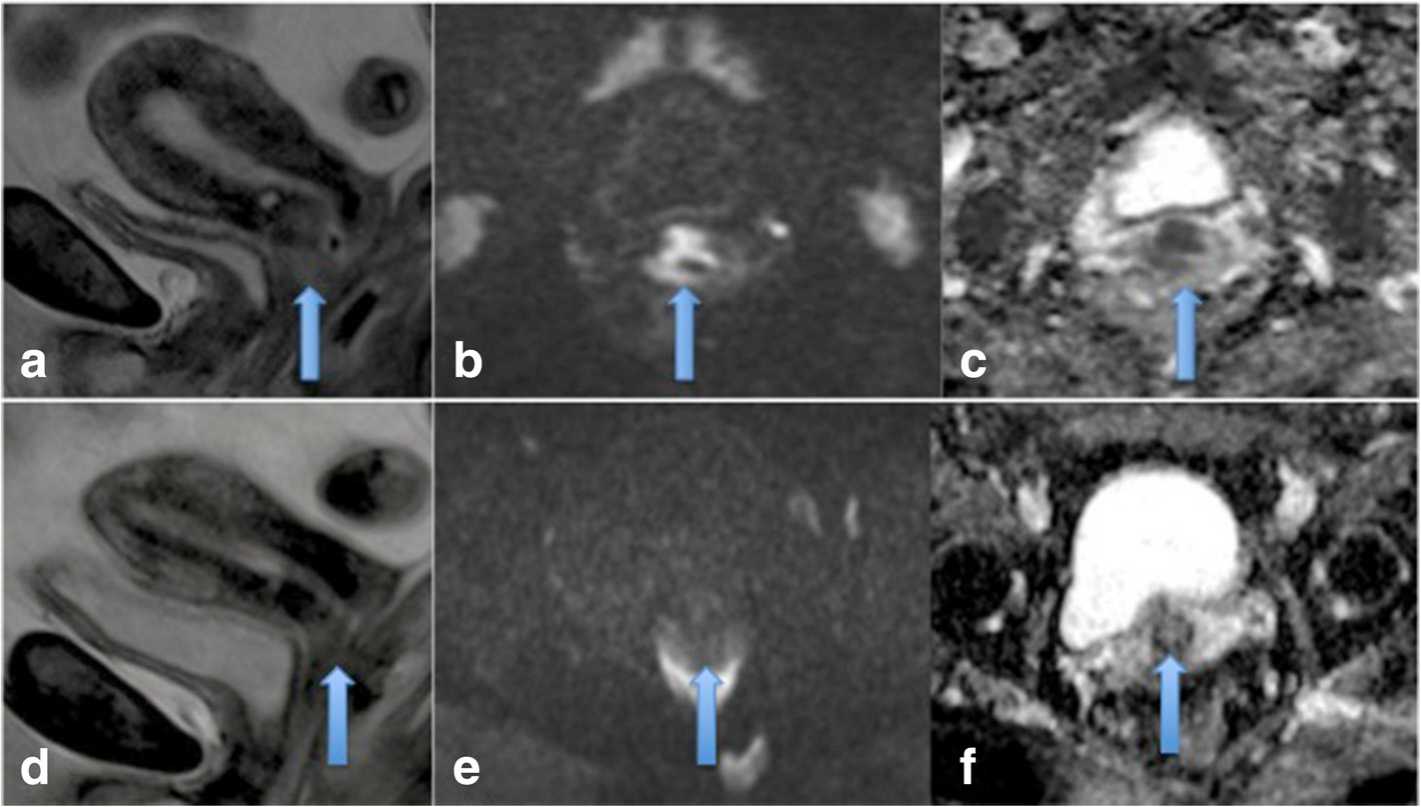
**Residual or recurrent disease and post treatment complete response.** Sagittal T2 image demonstrating an intermediate T2 signal intensity mass in the cervix which is either residual or recurrent disease **(a)**, axial DWI images through the tumour showing restricted diffusion, high signal on b1200 image **(b)** and low signal on the corresponding ADC map **(c)**. Post-treatment images **(d-f)** demonstrating no residual disease on T2 weighted sagittal images and axial DWI images.

**Figure 2:**
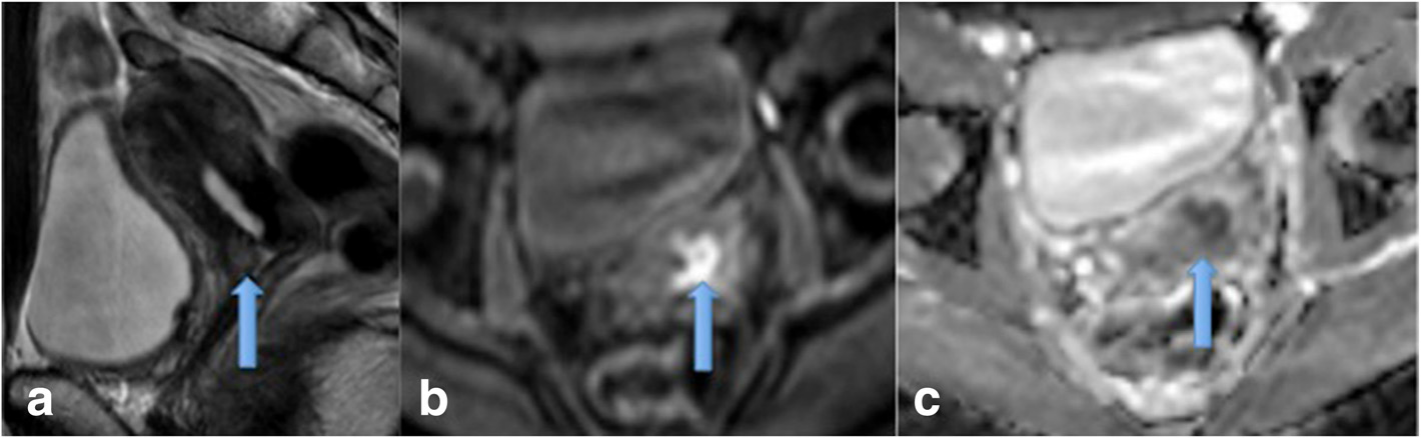
**Residual cervical disease.** Post treatment images showing a small area of residual disease in the anterior lip of the cervix on sagittal T2 MRI **(a)** and corresponding restricted diffusion **(b and c)**.

Cervical cancer is the leading gynaecological cancer worldwide and a significant proportion of patients will develop recurrent disease (30%) with a 64% 5 year survival rate [3]. Organ confined FIGO stage 1a/Ib disease is treated surgically and locally advanced disease is treated by chemoradiation. Imaging within three months of therapy is particularly challenging to interpret following radiotherapy as the tumour microenvironment is affected by hypoxia, granulation tissue and oedema and both residual disease and radiation fibrosis show increased T2 signal intensity [4] (see Figures 1 and 2). In this setting MRI is limited by false positive results, as demonstrated by Vincens et al, who correlated end of treatment MRI results with histopathological findings in patients with cervical cancer and found a sensitivity and specificity of 80% and 55% respectively for the detection of residual disease [5]. Developing an imaging biomarker that can pre-empt poor response to radiotherapy and detect residual disease and early recurrence with good accuracy would have profound prognostic implications.

The standard of care in early endometrial carcinoma is surgery with good outcomes owing to the early stage of presentation (80% of endometrial cancers are stage 1) [6]. However its incidence continues to rise with a 40% increase in the UK since 1993 [7]. The overall risk of recurrence is published at 13% and commonly occurs at the vaginal vault and pelvic lymph nodes but as there is a lack of consensus over optimal surveillance methods and patients may be asymptomatic, detection of recurrence can be challenging [8]. A reproducible biomarker to select those patients at risk of recurrence would similarly have great clinical impact.

Following endometrial cancer, ovarian cancer is the second most common gynaecological malignancy associated with high mortality rates due to late presentation [9]. Stage III disease is associated with a 5 year survival of 32-47% and following chemotherapy, relapse rates are high with a median progression free survival of 18 months [10]. 18 F-FDG PET/CT is the mainstay of imaging patients with suspected recurrence and performs better than CT and MRI and is particularly useful in detecting patients with irresectable disease [11]. MRI and CT perform less well than in primary staging due to the microscopic nature of peritoneal seeding and distortion of anatomy in the post-surgical state [12].

Results from the MRC OV05/EORTC 55955 trial concluded a lack of survival benefit in asymptomatic patients with biochemical relapse [13], however it remains important to develop imaging techniques to enable selection of symptomatic patients with early recurrence who may benefit from secondary cytoreduction which is associated with an increase in median survival [14].

### Functional imaging techniques

Changes in tumour microenvironment at a molecular level are known to precede morphological changes demonstrated on conventional MRI [15]. Dynamic contrast enhanced (DCE) MRI and diffusion weighted imaging (DWI) sequences reflect changes in oxygenation, perfusion and tissue physiology of the tumour microstructure and yield quantitative and semi-quantitative parameters which can potentially be used as a biomarker of tumour characteristics [2,16,17].

The ability to predict which cancers may respond to primary treatment provides an opportunity to manipulate treatment pathways. This allows the clinicians to individualise patient care limiting the use of unsuccessful treatment regimes with toxic side effects and high costs, whilst allowing other more suitable options to be instituted earlier [18]. The use of post therapy DCE-MRI in cervical and endometrial cancer may be able to predict which patients are at risk of early recurrence and therefore super select patients suitable for exenterative surgery.

#### DCE-MRI

DCE-MRI is a non-invasive technique which can assess tissue perfusion and oxygenation within the tumour microenvironment [19]. Abnormalities in tumour microvasculature and changes in perfusion can lead to hypoxia which is considered a major factor in response of tumour cells to radiation and in particular to radio-resistance [1,20]. The ability to evaluate the extent of hypoxia may be a useful predictive tool in identifying which patients may benefit from additional or more aggressive therapies.

Direct measurement of oxygenation of the tumour microenvironment is invasive, subject to sampling errors and several techniques have not been fully validated [21]. Imaging attention has been centred towards further evaluating DCE-MRI as a predictive perfusion biomarker. The ability to use routine gadolinium based contrast agents, most commercially available MRI scanners and analysis software allows DCE-MRI to be widely accessible. Moreover, the additional sequences are short adding only 10 minutes of scan time which is well tolerated by patients.

DCE-MRI involves intravenous injection of standard low-molecular weight paramagnetic intravascular contrast agents (typically gadolinium diethylene-triamine penta-acetic acid), either as a fast bolus pump injection or constant infusion. Serial image acquisitions are performed every few seconds over a length of a few minutes, the exact protocols and the region of interest depending on the tumour type being imaged.

The contrast agent reduces T1 longitudinal relaxation time of blood which increases signal intensity (SI) enhancement, of vascular structures on T1 imaging. When microcirculation of tumours is disrupted with angiogenesis and leaky capillary membranes, there is rapid accumulation of contrast agents and subsequent enhancement of tissues that is greater than that of similar normal tissue [2].

#### Semi-quantitative analysis

**Figure 3:**
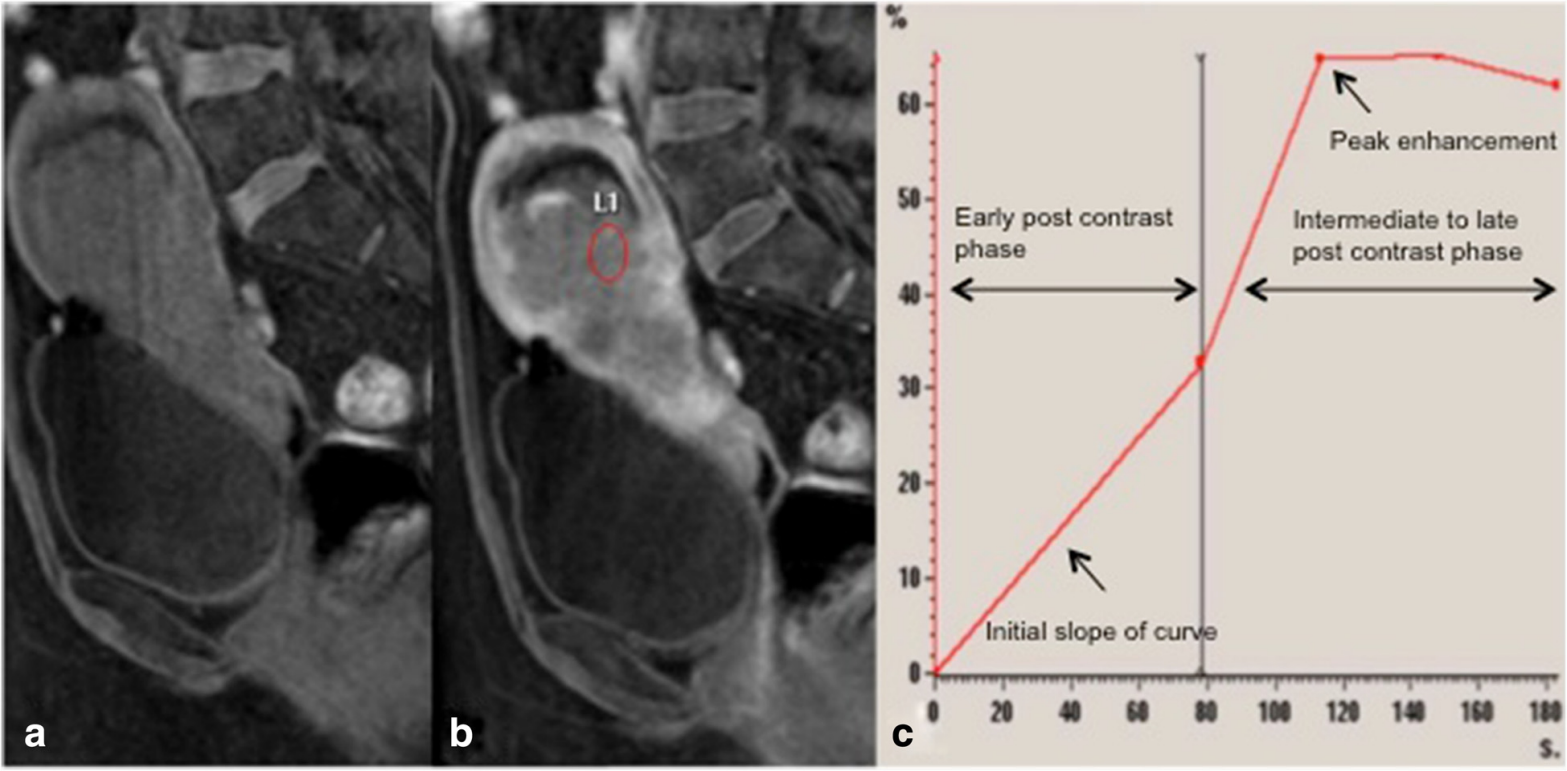
**Enhancing endometrial carcinoma and type 3 malignant curve. a** demonstrates the pre-contrast sagittal MRI through the uterus, following contrast at a 72 second acquisition, there is enhancement of endometrial soft tissue **(b)**, note that the tumour is less enhancing than the adjacent myometrium and invasion by the tumour can clearly be seen. A region of interest (red circle) has been placed over the tumour and a TCI created **(c)**, this shows a type 3 malignant curve.

DCE indirectly evaluates distribution of contrast by measuring tissue enhancement over time. The SI values are recorded before, during and after contrast administration and a dynamic time-signal intensity (TSI) curve is generated using a fixed region of interest (ROI) within the organ imaged (Figure 3).

The data that is commonly extracted from TSI curves includes: time to onset of enhancement, relative signal intensity (RSI), ratio of the maximum post-contrast SI to the pre-contrast SI which is a measure of the degree of enhancement, initial slope of the curve which is a measure of rate of enhancement and area under the curve which represents overall enhancement [22].

Table 1 summaries the adnexal, cervical and endometrial protocols used in the authors’ institution for both DCE and DWI-MRI for semi-quantitative analysis (Table 1).

**Table 1:** **Adnexal, cervical and endometrial MRI protocols for DWI and DCE-MRI at the authors institution**

**Sequence**	**Plane**	**FOV**	**Slice**	**Gap**	**Matrix**	**NSA**
**DWI 2b-values (0,1200)**	Axial	400	5	1	112 × 256	3
**DWI 3b-values (0,300,600)**	Axial (sagittal optional for endometrial cancer)	340	6	1	112 × 256	3
**T1 fat sat**	Adnexal:Axial oblique	280	4		224 × 256	8
**DCE 5 scans**						
**36secs each**						
**Scan time 3 mins**	Cervical and endometrial: Sagittal	280	4		208 × 256	6

#### Quantitative analysis

Whilst clinical evaluation can be obtained from semi-quantitative analysis, the data is not readily transferable between scanners and measurements are not an accurate assessment of the amount of contrast within the ROI [19]. To overcome these shortfalls, quantitative analysis is required. To perform quantitative analysis concentration of contrast is calculated within a ROI, deduced from analysing properties of T1 relaxation times pre and post-contrast. The National Cancer Institute has recommended that quantitative measurements, such as Ktrans (volume transfer constant between the plasma and the extracellular extravascular space) and IAUGC (the initial area under the gadolinium concentration–time curve], should be used as primary endpoints for DCE-MRI studies [23]. However quantitative analysis requires extensive support from dedicated MRI physicists and is labour intensive. Consequently, this has led to mainly the use of semi-quantitative DCE in clinical practice.

#### DWI-MRI

Functional imaging with DWI is a non-invasive technique which generates tissue contrast from differences in mobility of water molecules that occurs during an MR pulse sequence [17]. Information regarding the integrity of cellular membranes and tissue cellularity can be obtained [17,24]. Excellent contrast-to-noise ratio is achieved based on differences in cellularity between tumours, which usually return high signal, and normal tissue, which usually returns low signal. The signal intensity in DWI is derived from the intensity of MR pulse sequence, represented by a b-value. B- values are increased incrementally from low to high, typically ranging from 0-1200s/mm2. As the B-values increase, this increases the SI from tissues with high cellularity and hence the conspicuity and detection of disease is increased with suppression of bowel and background tissue, however this is at the expense of signal-to-noise ratio. High b-values between 800-1200s/mm2 are preferred for imaging the female pelvis to eliminate affects of T2 shine through from cysts, bladder and bowel [24,25].

Quantitative assessment of the tumour microenviroment can be derived from the apparent coefficient diffusion (ADC) which is generated from combining serial b-values [26]. Hence, DWI can be used to assess changes in tumour cellularity over time in response to treatment [16]. Acquisition of DWI and generation of ADC maps is simple, fast, and does not require intravenous contrast agents. ADC is a reproducible physical constant with advantageous features of a potential biomarker. ADC is exquisitely sensitive to changes in the water environment, therefore ADC values are affected by several factors from the hardware (scanner type and field strength) to the type of sequences used for example breath-hold, respiratory triggered or free-breathing [27]. These factors must be considered when interpreting changes in ADC values to ensure they are not the result of measurement error and reflect actual changes to the tumour [28]. Therefore in clinical practice and clinical trials the variability of ADC measurements should be minimised by *s*tandardization of b values, standardising imaging protocols and by consistent placement of serial ROI over time which is essential to ensure reproducibility of data.

There is great interest in use of ADC as a biomarker of early treatment response in several organs of the body, particularly breast, rectal and intracranial neoplasms with growing evidence in gynaecological malignancy, particularly cervical cancer [29,30].

## Clinical application of Functional MRI

### Cervical cancer

#### DCE-MRI as a predictor of radiotherapy response in cervical cancer

**Figure 4:**
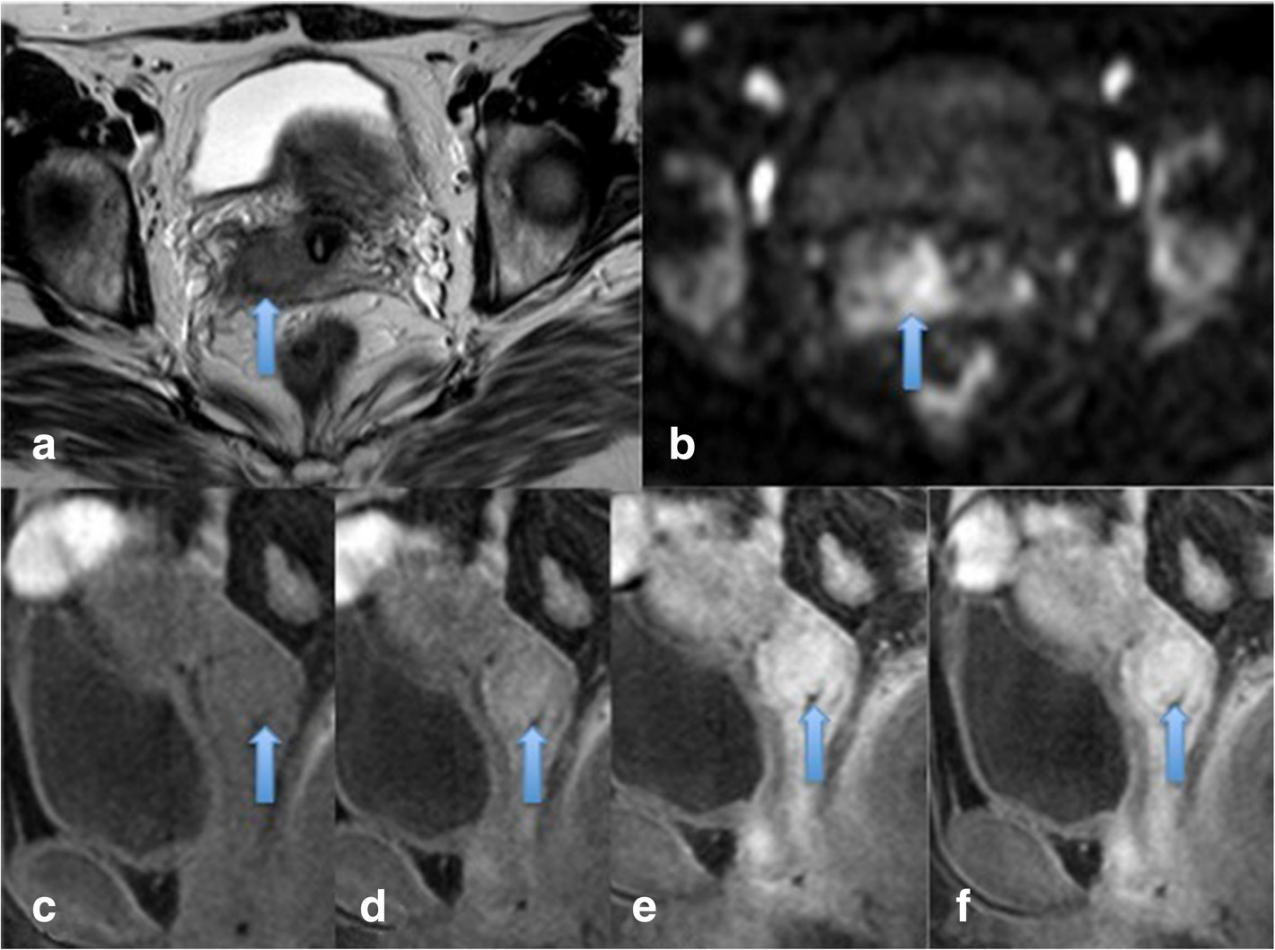
**Cervical carcinoma showing restricted diffusion and enhancement.** Axial T2 image showing a mass in the right lateral aspect of the cervix with parametrial invasion **(a)**, the mass demonstrates restricted diffusion on high b-values **(b)** and marked enhancement on DCE imaging (images **c-f**), indicating the tumour should respond well to treatment.

**Figure 5:**
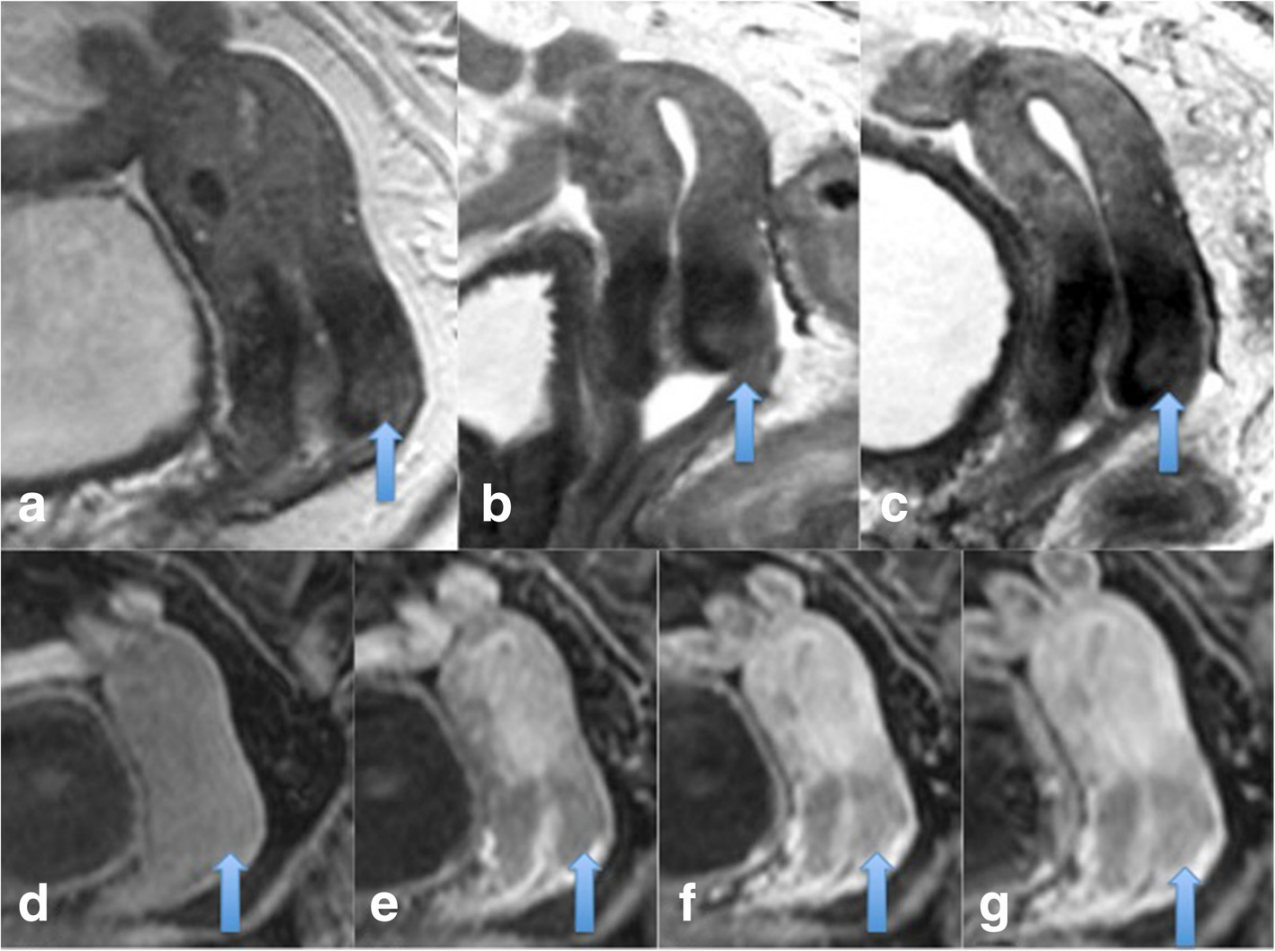
**Poorly enhancing cervical tumour indicating poor response to treatment.** Pre-treatment sagittal T2 MRI showing a tumour in the posterior cervical lip **(a)**, at the end of treatment there is recurrent disease **(b)** and 6 months later residual disease is present **(c)**. The pre-treatment DCE images are shown in figures **d-g** from pre-contrast to later contrast enhancement, minimal enhancement of the tumour is seen predicting the poor outcome to treatment observed.

Several studies have shown pre-treatment DCE-MRI parameters of high relative SI or peak enhancement are associated with tumour regression and local tumour control [19,21,31]. High perfusion before and during radiotherapy suggests increased vascularity and high tumour oxygenation, both associated with better response to treatment and therefore better prognosis. Mayr et al demonstrated an increase in tumour perfusion or relative SI early during radiation therapy for cervical cancer suggesting improved oxygenation of previously hypoxic cells making radiotherapy more effective in these tumours. More recently, the same group performed a longitudinal study evaluating perfusion at three time points throughout treatment in 98 patients, they found that if initial pre-therapy perfusion was low and subsequently changed to high perfusion early in therapy, a favourable outcome was obtained, but persistently low perfusion throughout treatment was associated with poor treatment response and poor prognosis [21,31] (see Figures 4 and 5). The definition of low perfusion varies between studies, Mayr et al analysed poorly perfused tumour subregions with voxel-by-voxel histogram analysis and quantified the areas as lower 10^th^ percentile of the signal-intensity curve (SI10%) – this was the value below which the lowest 10% of all tumour voxels fell and which differentiated favourable from unfavourable outcomes [31]. Yuh et al found that SI percentiles ranging from SI5%-SI20% provided overall best prediction with the single best predictor for primary tumour control was SI5% at 2-2.5 weeks of therapy [32]. In the authors institution qualitative assessment is also used to assess perfusion with low perfusion taken to be tumour enhancement less than normal cervical tissue and high perfusion taken to be tumour enhancement greater than cervical tissue.

Table 2 summarises pre-therapy DCE-MRI studies in cervical cancer and their key outcomes.

**Table 2:** **Pre-therapy DCE-MRI studies in cervical cancer and key outcomes**

**Authors**	**Number of patients**	**DCE-MRI parameters**	**Outcome**
Mayr et al 2010 [31]	98	Semi-quantitative RSI throughout treatment	● Persistent low perfusion associated with poor prognosis
			● Low perfusion which converts to high perfusion during treatment associated with favourable response.
Zahra et al 2009 [19]	13	Semi-quantitative and quantitative. Assessed at 3 time points, pre treatment, after 2 weeks of EBRT and in the last week of EBRT	● semi quantitative and quantitative DCE-MRI parameters correlate significantly with tumour regression at the end of EBRT.
			● Tumors with early peak SI, steeper slope for the TSI curve, and higher values for CER, Ktrans, and kep had more significant tumor regression and response to therapy
Mayr et al 1996 [21]	17	Semi-quantitative pre treatment and at 2 weeks EBRT.	● High pre-treatment SI and steeper slope on TSI curve correlated with lower incidence of local recurrence
			● High perfusion early in therapy is a favourable prognostic factor.
Yuh et al 2009 [32]	101	Semi-quantitative volume and perfusion in MRIs at 3 time points	● Tumor volume, mean SI, and SI% showed significant prediction of the long-term clinical outcome
Gong et al 1999 [33]	7	Semi-quantitative pre-treatment and at 2 weeks	● Mean and peak enhancement correlated with tumour regression
Yamashita et al 2000 [1]	36	Semi-quantitative	● Tumours with homogenous contrast enhancement correlated with good responders and poorly enhancing tumours with poor responders

#### Post-therapy DCE-MRI in predicting recurrence

**Figure 6:**
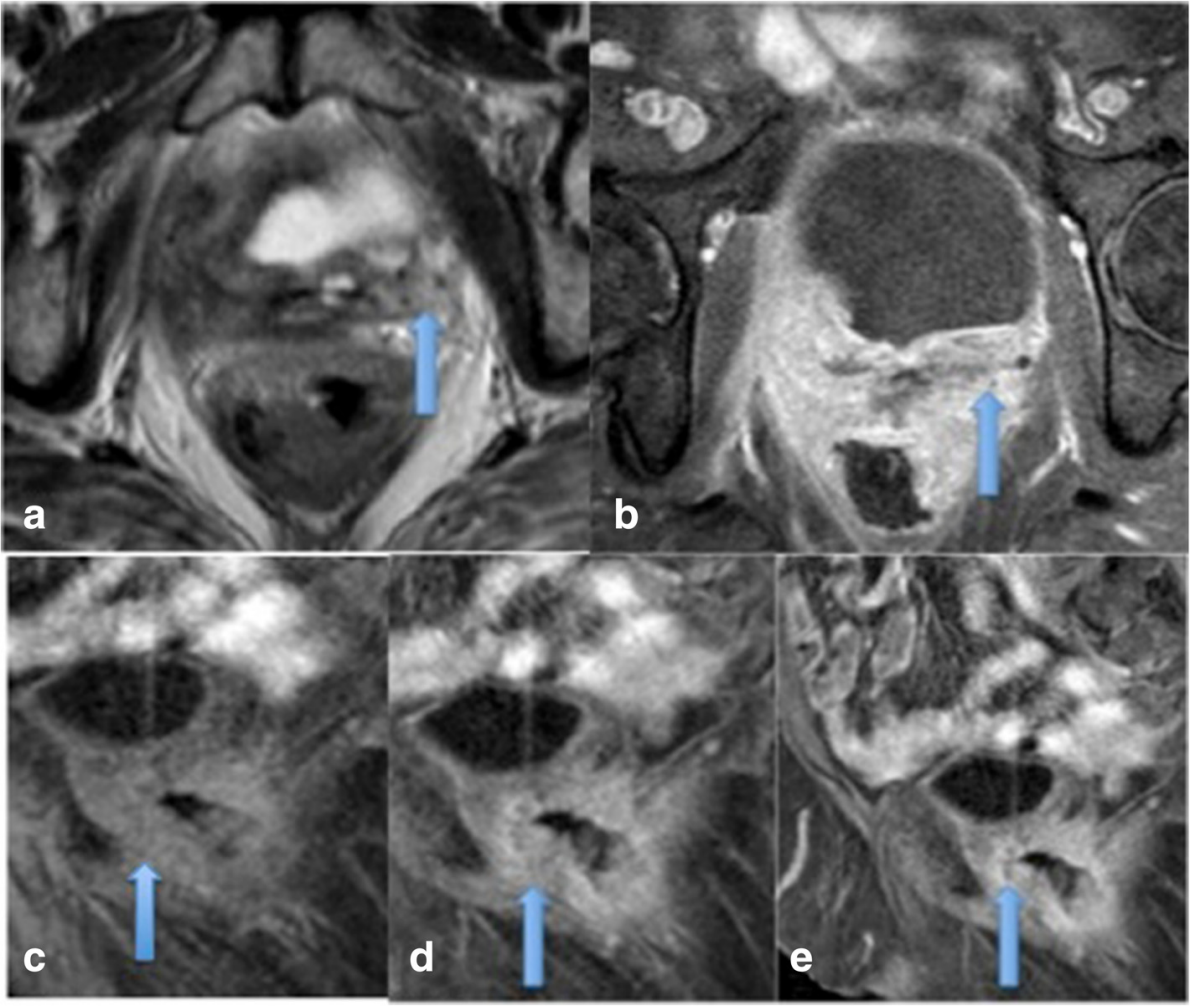
**Recurrent disease.** Recurrent mass at the left aspect of the vaginal vault after treatment for cervical cancer demonstrated on axial T2 images **(a)** and showing avid enhancement post contrast **(b)**. DCE images **(c-e)** showing strong early contrast enhancement.

At completion of therapy, persistent enhancement at the original cervical tumour site or in the post-surgical bed likely represents residual disease associated with increased risk of recurrence and poor survival. Post-therapy DCE-MRI can be used as a predictive tool to pre-empt or detect early residual disease and recurrences and thereby select patients suitable for exenterative surgery [18,34] (Figure 6).

In a small group of 10 patients with cervical cancer, Boss et al used a cut-off value of 6 seconds on the end of treatment DCE MRI to divide non-survivors and survivors. Early contrast enhancement of less than 6 seconds was associated with poor survival, but delayed contrast enhancement over 6 seconds reflected fibrosis and improved survival [35]. However further research into the ideal cut-off value in larger cohorts of patients is required to be of use in clinical practice.

#### DCE-MRI and tumour volume

Tumour size alone is an independent prognostic indicator in cervical cancer, reflected in the FIGO staging. In large cervical tumours it is thought areas of necrosis and poor vascularity contribute to tumour hypoxia and low contrast enhancement on DCE-MRI associated with poorer response to therapy. This contrasts with small highly vascular tumours which are more radiosensitive. However Mayr et al demonstrated that there was a lack of correlation between tumour volume and enhancement in intermediate sized tumours (between 4-6 cm) thought to reflect tumour heterogeneity [36]. The study found combining tumour volumetry and DCE enhancement variables help to refine the prediction of tumour response in this subgroup.

#### ADC as a potential biomarker of tumour response

A number of authors have shown following chemoradiation in cervical cancer the ADC value at the site of disease increases towards normal cervical ADC values, but does not reach that of normal cervical tissue, reflecting a favourable response to treatment but not necessarily differentiating post-treatment fibrosis from residual tumour [4,37]. This is thought to be due to effects of anti-cancer therapies causing tumour lysis, increased extracellular space which allows an increase in mobility of water molecules and diffusion [4]. Limitations of the published studies includes small numbers of patients analysed, the lack of a histological gold standard and also in the study by Chen et al, the ADC values were only evaluated in the first 3 months of completion of therapy. Before DWI can be used in routine monitoring of treatment, pre treatment threshold values for ADC need to be determined. Long-term studies evaluating the change in ADC values in different post-treatment stages are required. Table 3 summarises pre and post treatment ADC values in key studies in cervical cancer.

**Table 3:** **Pre and post treatment ADC values in cervical cancer in key studies**

**Author**	**No**	Pre-treatment ADC*	**Post treatment ADC**	**Percentage change**
Naganawa et al [37]	9	1.04 ± 0.17	1.48 ± 0.23	42%
Chen et al 2009 [4]	22	1.013 ± 0.094	1.436 ± 0.129	42%
Liu et al 2009 [38]	17	0.8 ± 0.08	1.08 ± 0.15	35%

*ADC [×10^−3^ mm^2^/s].

#### ADC as a biomarker of early response: mid-treatment

Harry et al demonstrated that there was a statistically significant correlation between mid-treatment ADC values and eventual MR and clinical response, there was a 21% increase between the pre-treatment ADC and mid-treatment values. No correlation was found between pre-therapy, post-therapy ADC values or baseline tumour size. The authors concluded in this small study of 20 patients, that mid-treatment ADC values may be a useful surrogate biomarker of treatment response in patients with advanced cervical cancer [39].

**Figure 7:**
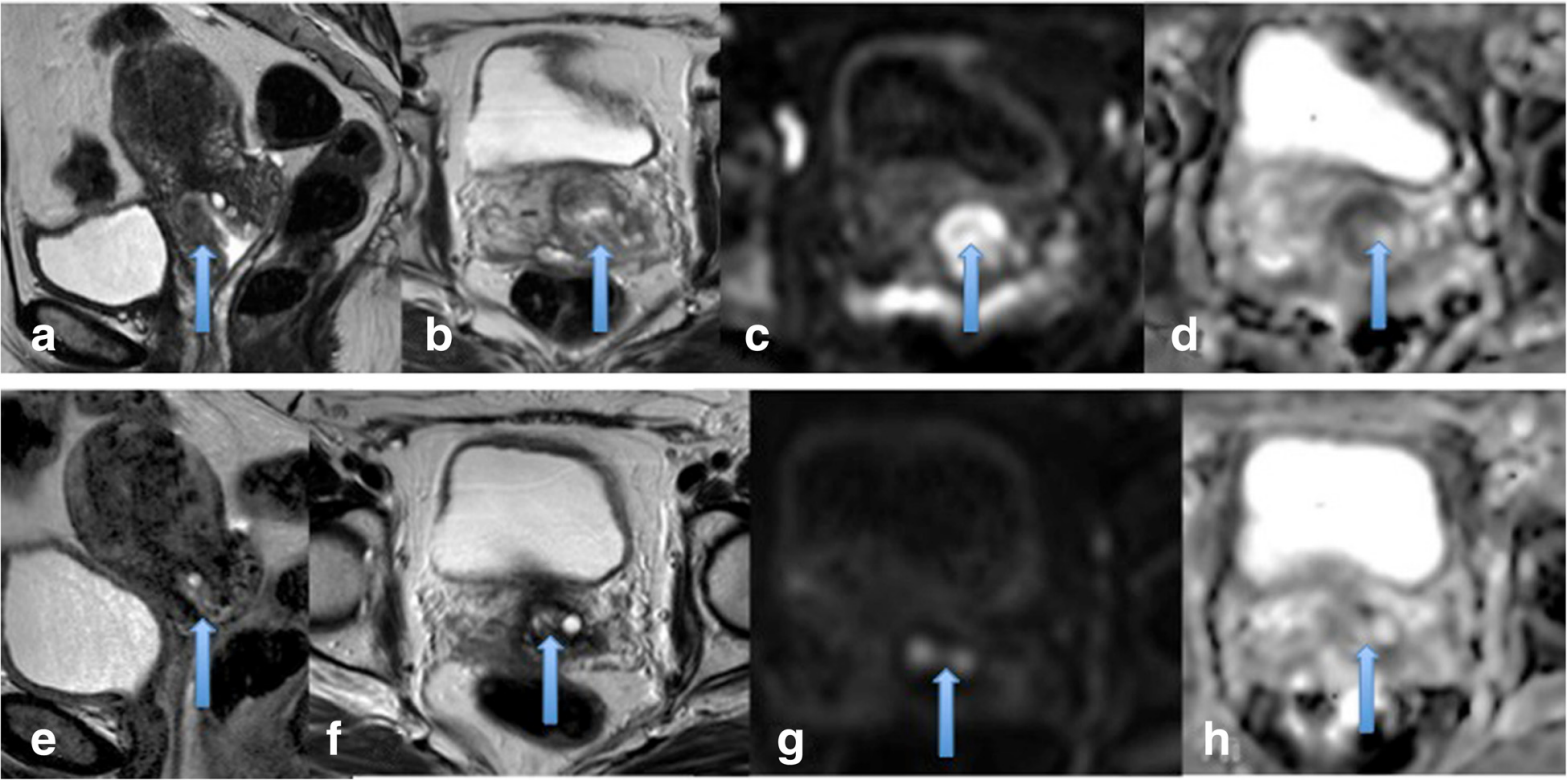
**Cervical carcinoma showing marked restricted diffusion and good response to treatment.** Pre treatment cervical cancer showing a mass on sagittal and axial T2 images **(a and b)** which demonstrated marked restricted diffusion with high signal on b1200 images **(c)** and low ADC **(d)**. Post treatment, no residual mass or restricted diffusion is present consistent with a good response to treatment **(e-h)**.

**Figure 8:**
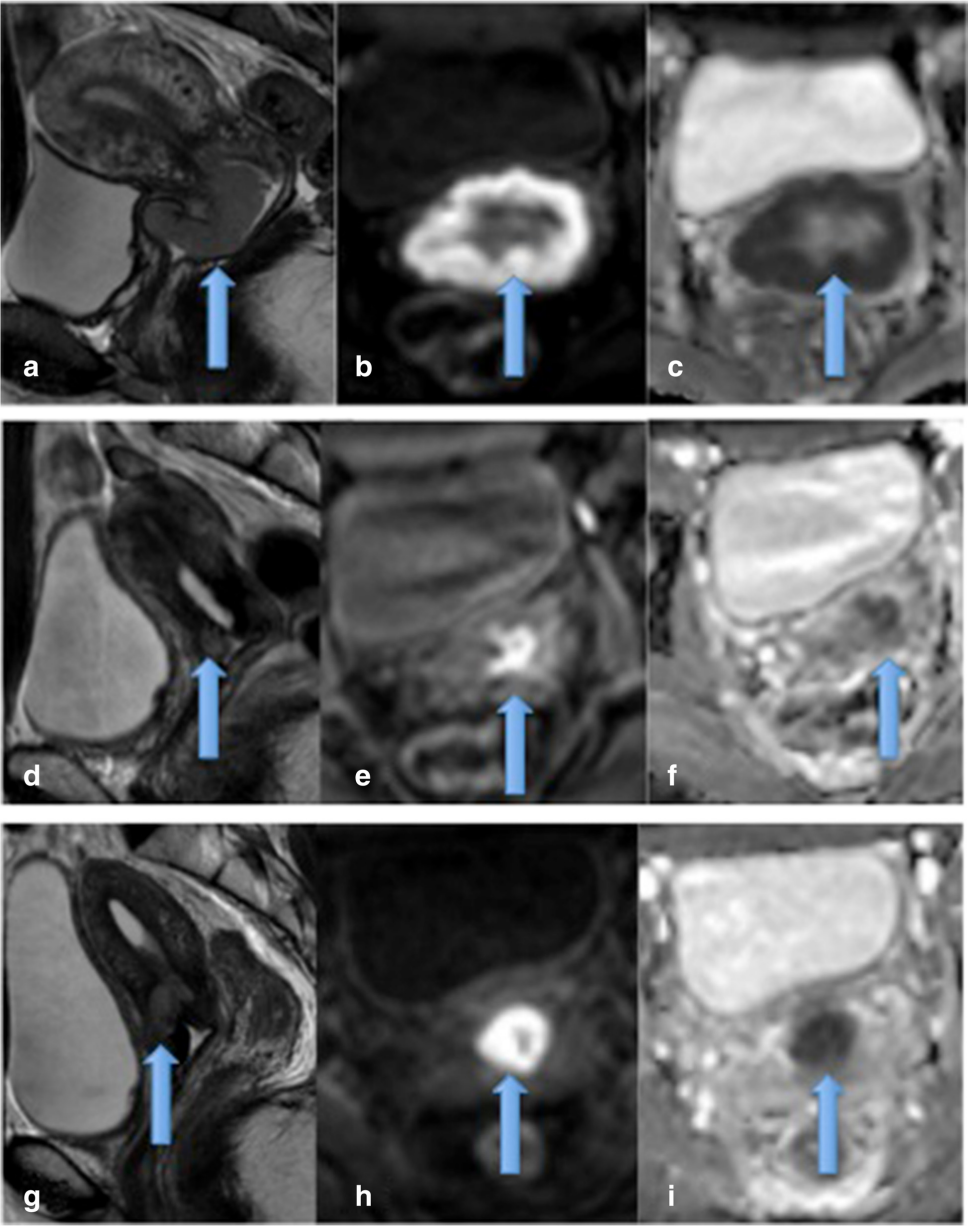
**Residual disease post treatment. a** demonstrates a pre-treatment large cervical mass on sagittal T2 imaging with marked restricted diffusion **(b and c)**. **d** demonstrates appearances mid-treatment with residual disease on T2 imaging and restricted diffusion **(e, f)**. At the end of treatment **g-i** demonstrates residual disease which has increased in size with corresponding increase in restricted diffusion.

Liu et al demonstrated two interesting findings in their study; pre- treatment ADC was lower in patients who went on to have a complete response (CR) compared to those in the partial response (PR) group. Secondly, the CR group mid-treatment ADC value increased rapidly and was higher than in the PR group supporting the fact that ADC values may predict those patients likely to be favourable responders [38]. Table 4 summaries ADC values at 3 time points in CR and PR subgroups (See Table 4) Figures 7 and 8.

**Table 4:** **ADC values at 3 time points in CR and PR subgroups in cervical cancer**

**Author**	**No**	**Baseline ADC**	**Mid-treatment ADC**	**S % change**	**Post- treatment ADC**	**% change**
**Liu et al**	15	CR 0.81 ± 0.08	1.25 ± 0.10	56%	__________	
		PR 0.93 ± 0.09	1.20 ± 0.17	29%	1.32 ± 0.23	42%

On review of the literature there is considerable overlap of the ADC values obtained pre, mid and post treatment in good and bad responders. Therefore the percentage change in ADC values at different time points during treatment may be of more clinical value than absolute ADC values. For example, in the work by Liu et al, complete responders demonstrated a 56% increase in pre and mid-treatment ADC values compared to only 29% change in partial responders, whilst Harry et al demonstrated a 21% increase between pre and mi-treatment ADC values. Larger studies are required to further establish clinical useful percentage parameters.

#### Association of ADC with survival and recurrence

In a follow up study by Somoye et al more recently, the authors were able to show that patients who had higher median mid-treatment ADC values had improved survival rates.

They found that survivors had a higher mid-treatment ADC (1.55 × 10^−3^ mm^2^/s) than non-survivors (1.36 × 10^−3^ mm^2^/s) which is a 14% difference. They found no evidence of a difference between survivors and non-survivors for pre-treatment or post-treatment ADC-values [15].

### Ovarian cancer

#### DCE-MRI in recurrent ovarian carcinoma

There are a small number of studies which have compared contrast enhanced MRI with second look laparotomies and have found comparable results. In one retrospective study of 68 patients with residual or recurrent tumour, DCE-MRI had a sensitivity of 90% and specificity of 88% compared with laparotomy (sensitivity 88%, specificity 100%) [40].

The authors concluded that serial Ca-125 in conjunction with MRI is more cost-effective than second look laparotomy and is associated with less morbidity. Therefore if a surgical approach is considered a pre-operative MRI is a potentially useful tool to aid surgical planning.

There is limited published literature assessing DCE-MRI in the response to treatment but one study by Sala et al looked at multi-parametric MRI in 22 patients treated for advanced ovarian disease and found that there was no change in the DCE parameters in ovarian, peritoneal and omental disease during treatment [41].

Contrast-enhanced MRI even without DCE is sensitive in the detection of subcentimetre peritoneal and serosal deposits due to the superior contrast resolution, particularly on delayed 5 minute acquisitions [42]. A pitfall of contrast enhanced MRI is that enhancement of the peritoneum and serosa is non-specific in the context of post-treatment complications including sepsis, fistulae and intra-operative chemotherapy [40,43].

#### DWI in ovarian and peritoneal disease

The sensitivity of contrast enhanced CT and MRI is comparable in the detection of metastatic ovarian disease. Detection of peritoneal disease is difficult due to poor contrast resolution between disease implants and adjacent bowel and surrounding structures. Because DWI demonstrates high contrast-to-noise ratio, detection of small volume subcentimetre disease is improved. As mentioned earlier, the DWI images should always be reviewed with the ADC maps and anatomical sequences to exclude T2-shine through, for example fluid in simple cysts will be high signal on T2 images and this can ‘shine through’ onto the DWI and ADC maps, if the sequences are not reviewed together then lesions may be interpreted as having restricted diffusion (which should appear as low signal on ADC maps). Knowledge of structures which may appear bright on DWI imaging, but are not disease related is important to avoid overcalling disease, for example reactive lymph nodes and bowel mucosa which have high cellularity [6]. When combined with conventional anatomical MR sequences, DWI improves accuracy to 84-88% on a per-lesion basis, while MRI alone is 52-72% and DWI alone is 71-81%, but this has yet to be correlated with a clinical impact [44]. A study by Low et al demonstrated an increase in detection of peritoneal deposits by 21% and 29% in 169 patients using single breath-hold DWI [45].

In one small study of 42 patients with recurrent ovarian or peritoneal cancer the ADC values during chemotherapy were analyzed and the authors found that in responders, the ADC values of lesions increased after cycle 1 and 3 compared with non-responders where no parameter changed significantly. Furthermore the pre-treatment ADC were not predictive of response to treatment. The authors concluded that quantitative DW-MRI may aid early monitoring of treatment efficacy [46].

In the same study by Sala et al quoted earlier the authors found that the baseline ADC of ovarian lesions, peritoneal lesions and omental cake differed significantly suggestive of biologic heterogeneity of disease. They also found that post-treatment the ADC of ovarian lesions increased significantly in responders where as there was no change in ADC in non-responders. The authors concluded that ADC values may be useful response markers whereas no significant change was seen in DCE or spectroscopy parameters [41].

### Endometrial carcinoma

#### DWI-MRI in predicting recurrence

There is limited literature evaluating DWI-MRI in predicting recurrence in endometrial cancer. Multiple important prognostic factors have been documented including age, stage, histology, depth of myometrial invasion and lymph node metastases however there are no biomarkers currently available to predict prognosis [6].

One study by Nakamura et al looked at 111 patients who underwent surgery for endometrial cancer. The authors found that there was a significant correlation between pre-treatment ADC values and FIGO stage, depth of myometrial invasion, cervical involvement, lymph node metastasis, peritoneal cytology and tumour size. In particular lower ADC values were associated with grade 1 histological classification compared with grade 3, and low ADC values were associated with disease free survival compared with patients with higher pre-treatment ADC values. No association was found with overall survival, but the authors concluded low pre-operative ADC could predict which patients have a low risk of recurrent disease [47].

### Future directions

A significant limitation of the current studies is the small number of patients sampled and the lack of a quantitative gold standard. Although quantitative analysis is desirable and recommended in guidelines published by the National Institute of Cancer for DCE-MRI, this is labour intensive requiring MRI physicist support. ADC as a quantitative, reproducible predictive tool shows great promise but also needs standardisation of b-values between institutions and further research into identifying threshold values. Percentage change in ADC values at various treatment points may serve as a better indicator of response than absolute values. In cervical cancer long-term studies evaluating differences in ADC values in the post-therapy setting over a longer time period may help to overcome the challenge of differentiating residual disease from fibrosis. There is a lack of published data evaluating functional imaging in both recurrent endometrial and ovarian cancer. The role of whole body MRI and PET-MRI are currently limited to the research setting but these modalities may have added benefit to the assessment of pelvic gynaecological malignancy due to the exquisite combination of high resolution anatomical and functional images. Further research into the correlation of early disease detection with long term survival and disease free morbidity is required to assess the clinical utility of advanced functional imaging.

## Conclusion

The majority of published data has evaluated functional MRI and cervical cancer with promising results to date. Some limited studies have shown added value of functional MRI in recurrent endometrial and ovarian cancers. Given that both DCE-MRI and DWI-MRI are non-invasive, readily accessible and without ionising radiation, there are advantages in being able use these techniques to further individualise and benefit patient care.
